# Open-door policy in urban acute wards: results and implementation experiences from a five-year staff co-created in-clinic RCT

**DOI:** 10.1192/j.eurpsy.2025.120

**Published:** 2025-08-26

**Authors:** N. Kunøe

**Affiliations:** 1 The Lovisenberg Diaconal Hospital; 2 The University of Oslo, Oslo, Norway

## Abstract

**Abstract:**

Open-door policy is a WHO-recommended framework to maximise safety, prevent coercion, and enhance recovery-based practices during admission to mental health wards. This talk will describe the open-door policy intervention, present results from our 1-year RCT and preliminary 5-year results from the Lovisenberg Open Acute Door Study (LOADS). LOADS is a 5-year in-clinic dual RCT study of open-door policy co-created with user representative and staff in our inner-city acute mental health wards in Oslo, Norway. By combining random allocation, effectiveness outcomes and implementation research, LOADS aims to address the need for faster development of clinically relevant knowledge in mental health.

**Disclosure of Interest:**

None Declared

